# Interpreting Viral Associations in Neurodegenerative Diseases

**DOI:** 10.3390/ijms27188185

**Published:** 2026-09-15

**Authors:** Anna Karin Hedström

**Affiliations:** Department of Clinical Neuroscience, Karolinska Institutet, 171 76 Stockholm, Sweden; anna.hedstrom@ki.se

**Keywords:** Epstein–Barr virus, multiple sclerosis, Alzheimer’s disease, Parkinson’s disease, amyotrophic lateral sclerosis, herpesviruses, human pegivirus, antiviral immunity, viral reactivation

## Abstract

Viral infections have been associated with multiple sclerosis (MS), Alzheimer’s disease (AD), Parkinson’s disease (PD), and amyotrophic lateral sclerosis (ALS), but the associations may reflect different relationships to the disease process. This review evaluates evidence for viral involvement in disease initiation, modification of established disease, impaired viral control secondary to disease or treatment, and incidental detection. The strongest temporal evidence concerns Epstein–Barr virus (EBV) and MS. Prospective data place EBV seroconversion before clinical onset and the first observed increase in serum neurofilament light chain, while mechanistic studies link EBV infection, B-cell biology, and CNS-directed immunity. However, evidence that ongoing EBV activity modifies established MS remains limited. In AD, experimental studies support interactions between herpesviruses and AD-associated proteins, while the reduced incidence of all-cause dementia after herpes zoster vaccination suggests that viral or immunological pathways may be modifiable, without establishing that a specific herpesvirus initiates AD. Viral associations in PD rely mainly on epidemiological, experimental, and postmortem findings. Human pegivirus detection in a subset of PD brains remains a candidate association requiring independent confirmation and evidence of biological activity. Evidence in ALS is similarly limited. Enterovirus detection has been inconsistent, and altered human endogenous retrovirus K (HERV-K) expression in postmortem tissue does not establish an acquired viral infection. Stronger inference across these diseases will require longitudinal studies relating viral activity and antiviral immunity to subsequent disease-related changes, together with intervention studies that document the intended effect on the implicated viral process and separately assess subsequent disease risk or progression.

## 1. Introduction

Viral infections have long been implicated in chronic neurological diseases, including multiple sclerosis (MS), Alzheimer’s disease (AD), Parkinson’s disease (PD), and amyotrophic lateral sclerosis (ALS), but the supporting evidence differs markedly between diseases [1,2,3]. The evidence ranges from prospective observations placing infection before detectable disease-related changes to serological and epidemiological associations, experimental models, and viral detection in cerebrospinal fluid or postmortem brain tissue.

Determining what a viral association represents requires placing infection or viral activity in relation to the biological course of the disease. Seropositivity at diagnosis confirms previous infection but cannot determine whether primary infection preceded the onset of the underlying disease process. In established disease, increased viral load, viral shedding, or reactivation may reflect impaired viral control caused by the disease or its treatment. Detection of viral nucleic acids or proteins in the central nervous system (CNS) does not by itself demonstrate replication or a contribution to disease [4,5]. Conversely, failure to detect the virus in the CNS does not exclude an effect mediated by peripheral infection or the host response [1].

The clearest temporal evidence concerns Epstein–Barr virus (EBV) and MS. In a large prospective study, EBV seroconversion preceded both MS onset and the first observed increase in serum neurofilament light chain among individuals who subsequently developed MS [6]. Findings in AD and PD require different interpretations, as illustrated by the association between herpes zoster vaccination and reduced dementia risk [7] and the detection of human pegivirus in a subset of PD brains [8]. This review examines what can be concluded from viral associations in MS, AD, PD, and ALS, using EBV in MS as the principal model and findings in the other diseases as contrasting examples.

## 2. Literature Identification and Selection

This narrative review was informed by targeted searches of PubMed and Web of Science conducted through 31 August 2026, without a lower date limit. Search terms combined each neurological disease (MS, AD or dementia, PD, and ALS) with terms for individual viruses, viral infection, serology, reactivation, antiviral treatment, and vaccination. Reference lists of relevant systematic reviews, meta-analyses, and primary studies were also examined.

English-language, peer-reviewed human studies were selected when they provided temporal, epidemiological, virological, or intervention evidence relevant to the interpretive framework. Experimental studies were included when they provided mechanistic context for human findings. Priority was given to prospective studies, systematic reviews and meta-analyses, large population-based studies, randomized or quasi-experimental studies, and studies using direct viral measurements. Small studies were retained when they provided otherwise unavailable tissue-based evidence. The review was narrative and intended to evaluate contrasting forms of evidence, not to provide an exhaustive systematic review of all reported virus–disease associations.

## 3. Interpreting Viral Associations

**Figure 1:**
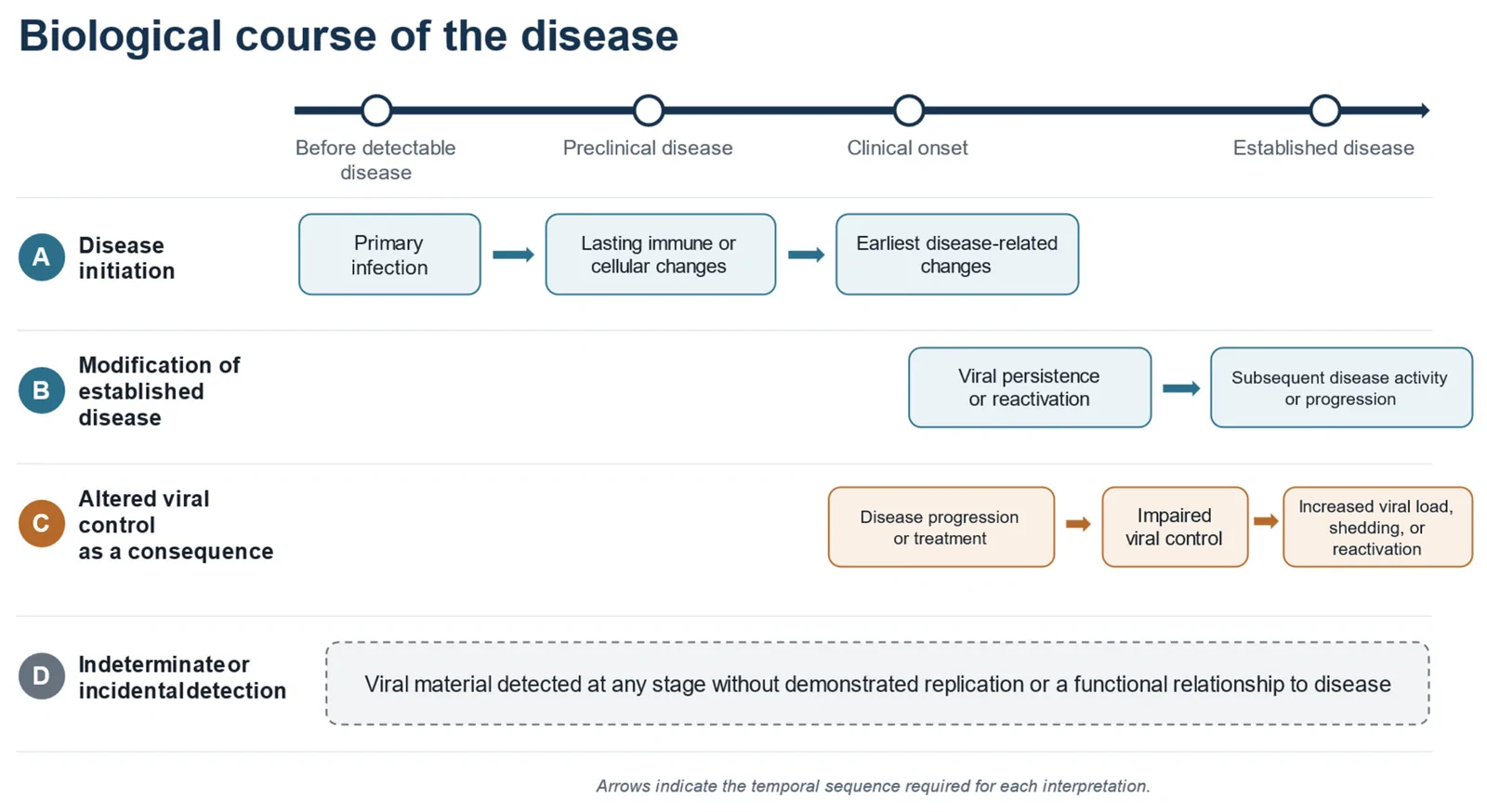
Possible roles of viral infection across the biological course of chronic neurological disease. An infection may contribute to disease initiation if it precedes the earliest detectable disease-related changes. During established disease, viral persistence or reactivation may precede and influence subsequent disease activity or progression. Disease progression or immunomodulatory treatment may instead impair viral control and increase viral load, shedding, reactivation, or detection. Viral material may also be detected without evidence of replication or a functional relationship to the disease. The arrows indicate the temporal sequence required for each interpretation.

The role suggested by a viral association depends on its timing in relation to the disease process. An infection that contributes to initiation may no longer be required once a self-sustaining pathological process has developed. The same virus may instead remain active and influence the subsequent disease course or become reactivated because the disease or its treatment has impaired viral control. Evidence for initiation and disease modification must therefore be assessed separately. Figure 1 summarizes these alternative interpretations and the temporal sequence required to support each.

Virus–disease associations must also be considered in relation to host susceptibility and other exposures. Age, sex, ethnicity, family history, genetic susceptibility, and environmental exposures may affect viral exposure, the host response, the underlying risk of neurological disease, or combinations of these. They may therefore confound observed associations or modify the effect of infection. In MS, the human leukocyte antigen (HLA)-DRB1*15:01 allele may modify the consequences of EBV infection by shaping the presentation of myelin-derived peptides by infected B cells [9]. In AD, prospective cohort data suggest that apolipoprotein E (APOE) ε4 may modify the association between serological markers interpreted as frequent herpes simplex virus (HSV)-1 reactivation and disease risk [10]. These examples suggest that host susceptibility can influence the consequences of viral infection. However, whether such effect modification contributes to variation in findings across populations remains unclear, and interactions with environmental exposures have been little studied. Evidence for an initiating role is strongest when infection precedes the earliest measured disease-related changes. Prospective cohorts with samples collected before symptoms are particularly informative when primary infection or seroconversion can be related to later biomarkers and disease onset [11,12,13]. Seropositivity establishes previous infection but does not determine when it occurred. Persistence of the virus at diagnosis is not required, since an initiating infection may induce immunological or cellular changes that continue after viral activity has ceased.

A disease-modifying effect requires viral activity or a defined virus-associated host response to precede a subsequent change in an established disease process. Repeated measurements are needed to relate changes in viral activity to inflammatory activity, imaging findings, disease-associated biomarkers, cognitive decline, or disability progression. Acute infections may temporarily worsen neurological symptoms without altering the longer-term disease trajectory [14]. Associations observed after diagnosis may also reflect effects of age, comorbidity, disease severity, or treatment on viral control [15,16]. When increased viral load, viral shedding, or reactivation follows disease progression or the introduction of immunomodulatory treatment, the findings instead support impaired viral control as a consequence of disease or treatment.

## 4. Methodological Considerations

Postmortem studies are vulnerable to both selection and detection bias. Brain donors may differ from the source population in disease severity, comorbidity, treatment, and terminal illnesses, which may affect viral detection or inflammatory signals. Comparisons may also be affected by differences between cases and controls in postmortem interval, brain region, tissue preservation, nucleic acid quality, the number of regions examined, and sequencing depth. Since viral nucleic acids are usually present at low abundance, analytical thresholds, misclassification of sequencing reads, and contamination can have a substantial influence on the results [5,17]. Findings should therefore be reproduced in independently collected and separately processed tissue and confirmed by a second method, together with cellular localization and, where possible, markers of viral replication.

Viral reactivation is also difficult to classify. Seropositivity and single antibody measurements do not establish reactivation, while isolated polymerase chain reaction (PCR) positivity may reflect transient shedding or residual viral material without demonstrating sustained or disease-relevant viral activity. Conversely, a negative sample does not exclude intermittent reactivation. Repeated quantitative measurements, predefined criteria for reactivation, and similar sampling schedules in cases and controls are needed to reduce misclassification and differential detection.

Vaccination studies are susceptible to healthy-vaccinee bias and residual confounding because vaccinated and unvaccinated individuals often differ in health, socioeconomic circumstances, healthcare use, and preventive behavior. Frailty and competing mortality may also affect both vaccination and the likelihood of receiving a dementia diagnosis. Natural experiments based on eligibility rules reduce several of these biases but do not identify the biological pathway responsible for an observed association [7]. Protection may result from prevention of the targeted infection or reactivation, adjuvant effects, or broader changes in immunity. Observational studies of antiviral treatment additionally require consideration of treatment selection and confounding by indication. Experimental studies can establish that a virus or antiviral immune response produces disease-relevant cellular effects, but not whether the same mechanism operates in human disease. Intervention studies are more informative when they verify that the intervention affected the proposed viral process and then assess a subsequent disease-related outcome. A change in viral activity alone does not establish an effect on the neurological disease. A proposed viral role is most persuasive when temporal, virological, clinical, and mechanistic evidence supports the same interpretation.

## 5. Epstein–Barr Virus and MS

Almost all individuals with MS have previously been infected with EBV, and both infectious mononucleosis and high antibody levels against EBV nuclear antigen 1 (EBNA1) are associated with increased MS risk [18,19]. The most informative evidence comes from a longitudinal cohort of more than ten million US military personnel. Among individuals who were initially EBV-seronegative, the risk of MS increased 32-fold after EBV seroconversion, while no corresponding increase was observed following infection with other viruses, including cytomegalovirus. Serum neurofilament light chain increased only after EBV seroconversion in individuals who later developed MS [6]. These findings place EBV infection before the first observed evidence of neuroaxonal injury and make reverse causation an unlikely explanation for the association. Neurofilament light chain does not capture the earliest immunological changes, and the precise interval between EBV infection and initiation of the MS disease process remains uncertain. Nevertheless, the combined evidence strongly supports an important role for EBV infection in MS development. Its high prevalence in the general population also shows that infection alone is insufficient and that additional genetic, environmental, and immunological factors determine who develops MS.

EBV establishes lifelong infection in the B-cell compartment, providing several possible links to MS pathogenesis [1]. Molecular mimicry may allow antiviral responses to recognize structurally related CNS antigens. Clonally expanded B cells isolated from the cerebrospinal fluid of people with MS have been shown to produce antibodies that bind both EBNA1 and the CNS protein GlialCAM, and immunization with EBNA1 aggravated disease in an experimental model [20]. Other studies have identified mechanisms involving antigen presentation by EBV-infected B cells. EBV infection can reprogram the transcriptome and immunopeptidome of B cells, promoting the presentation of myelin-derived peptides on MS-associated HLA-DR15 molecules [9]. In an experimental model, expression of the EBV protein latent membrane protein 1 allowed B cells that had captured myelin antigen in the CNS to survive and induce demyelinating lesions [21]. These studies identify plausible ways in which EBV infection, B-cell biology, and genetic susceptibility may converge. They do not establish which mechanism predominates in human MS or whether continued viral activity is required once the autoimmune response has been established.

Whether EBV also sustains inflammation after MS onset remains uncertain. EBV-infected B cells have been reported in meningeal infiltrates and MS lesions, but these findings have not been consistently reproduced [22,23]. Differences in tissue preservation, sampling, and detection methods may contribute to the discrepant results. Even confirmed detection of EBV-infected cells would not show that they maintain compartmentalized inflammation or promote lesion expansion. No longitudinal study has demonstrated that EBV activity precedes the development of chronic active lesions or subsequent disability progression.

EBV-specific antibody and T-cell responses also differ between people with MS and controls after disease onset, but their relation to viral activity is uncertain. Higher anti-EBNA1 antibody levels have been associated with markers of disease activity or worse clinical outcomes in some studies, although these associations have not been consistent [24,25,26]. Antibody concentrations are not direct measures of viral load or reactivation and may reflect long-standing differences in the host response influenced by genetic factors, disease duration, and treatment. A recent study found increased CD4-positive T-cell responses to late lytic EBV antigens in untreated people with MS. These responses decreased after anti-CD20 treatment, accompanied by elimination of detectable EBV shedding in saliva [27]. The findings support an altered immune response to the EBV lytic cycle in MS but do not establish that ongoing viral replication drives disease activity or progression. The clinical efficacy of B-cell-depleting therapies and the accompanying changes in EBV-specific responses are compatible with a role for EBV-infected B cells, but do not distinguish depletion of the viral reservoir from other effects of removing B cells from the immune response.

Prospective seroconversion data support an upstream role in MS development, and mechanistic studies provide biological support for this interpretation [6,9,20,21]. However, evidence that ongoing EBV activity modifies the course of established MS remains limited.

## 6. Viral Associations in AD and Dementia

Several herpesviruses have been investigated in relation to AD, particularly HSV-1, varicella-zoster virus (VZV), and human herpesviruses (HHV), including HHV-6A and HHV-7 [2]. Primary infection with these viruses generally occurs decades before the clinical onset of AD, and proposed disease-relevant effects have therefore often involved viral persistence or reactivation. Human evidence has come mainly from serological studies, health-record studies of clinically recognized infection, and viral detection in postmortem brain tissue [2,28,29]. These approaches have not established whether viral reactivation precedes AD pathology or becomes more frequent as a consequence of disease-related changes in viral control.

Postmortem studies have produced inconsistent findings. A multiscale analysis of several AD cohorts reported increased abundance of HHV-6A and HHV-7 and associations between viral abundance and molecular networks implicated in AD [28]. Subsequent analyses using RNA sequencing and direct detection of viral DNA across independent brain collections found little specificity of HHV-6 for AD [29]. Differences in tissue region, sample handling, sequencing depth, and analytical methods may contribute to these discrepancies. The low abundance of viral reads makes these analyses particularly sensitive to analytical thresholds, read misclassification, and contamination [5,17,29]. Positive findings therefore require confirmation by independent methods in separately processed samples. Even when confirmed, the presence of herpesvirus nucleic acids in brain tissue does not identify their cellular source, demonstrate viral replication, or show that the virus influenced the disease.

Experimental studies provide several mechanisms through which herpesvirus infection could interact with AD-related pathology. Amyloid-β has antimicrobial and antiviral properties, and exposure to HSV-1 and HHV-6 was shown to accelerate amyloid-β deposition, resulting in the entrapment of viral particles in cell culture and mouse models [30]. Other studies have linked HSV-1 infection to altered amyloid processing, tau phosphorylation, and microglial activation, although effects on amyloid pathology have differed between experimental models [31,32,33]. These findings support interactions between herpesvirus infection and AD-associated proteins but do not determine whether infection initiates the pathological process in humans. Similar interactions could occur after amyloid or tau pathology is already established.

Evidence concerning cytomegalovirus (CMV), another persistent herpesvirus, has mainly come from seroepidemiological studies. In a longitudinal community cohort, CMV seropositivity was associated with incident AD and faster cognitive decline [34]. A meta-analysis of seven studies also reported higher odds of AD among CMV-positive participants, although the study designs and estimates varied [35]. A broader meta-analysis likewise found a modest association, but the pooled estimate was not robust to the removal of individual studies [36]. Since CMV is usually acquired long before late-life cognitive decline and seropositivity varies with age, socioeconomic conditions, and immune function, these findings do not establish that CMV reactivation preceded AD pathology. Repeated measures of viral activity and CMV-specific immunity linked to subsequent AD biomarkers would be needed to support this interpretation.

Antiviral treatment has also been examined in observational studies. In a Swedish registry-based matched cohort, antiviral treatment was associated with a lower risk of dementia, whereas clinically diagnosed HSV or VZV infection without antiviral treatment was associated with a higher risk [37]. Matching was limited to birth year and sex, and differences in infection severity, comorbidity, treatment selection, and healthcare use could have contributed to these associations.

The VALAD randomized trial directly tested whether valacyclovir improves clinical outcomes in HSV-seropositive patients with established AD. A total of 120 participants with early symptomatic AD and HSV-1 or HSV-2 seropositivity received valacyclovir or placebo for 78 weeks. Valacyclovir did not slow cognitive or functional decline or alter amyloid or tau imaging outcomes, and cognitive worsening was greater in the valacyclovir group [38]. These findings argue against valacyclovir as a treatment for established AD in individuals selected solely by HSV seropositivity. They do not exclude a role of HSV earlier in the disease process.

By contrast, herpes zoster vaccination provides an opportunity to examine whether an intervention before dementia diagnosis can modify subsequent risk. In a natural experiment based on a date-of-birth eligibility threshold in Wales, receipt of the live-attenuated zoster vaccine reduced the probability of a new dementia diagnosis by approximately 20% over seven years [7]. The design limits many of the differences in health and healthcare behavior that affect conventional comparisons between vaccinated and unvaccinated individuals. The outcome was dementia of any type, and the study did not establish whether the effect resulted from prevention of VZV reactivation. The authors identified both reduced VZV reactivation and VZV-independent immunological effects as plausible mechanisms.

Additional observational findings support a role for VZV reactivation. A large analysis of electronic health records found a higher dementia risk after recurrent than single episodes of herpes zoster. The reduction in dementia risk after vaccination varied with vaccine dose and waned in parallel with protection against herpes zoster [39]. These patterns are consistent with VZV reactivation contributing to dementia risk but remain based on clinically recorded exposures and outcomes. Residual differences between comparison groups, incomplete detection of subclinical reactivation, and changes in healthcare use cannot be fully excluded.

Evidence for broader vaccine effects further complicates the interpretation. The recombinant zoster vaccine contains the AS01 adjuvant, and vaccination against either herpes zoster or respiratory syncytial virus with an AS01-adjuvanted vaccine has been associated with a lower subsequent risk of dementia [40]. Similar associations for vaccines targeting different viruses raise the possibility that part of the observed protection is mediated by the adjuvant or by broader effects on innate and adaptive immunity. They do not exclude an additional benefit from preventing VZV reactivation.

The vaccination studies are consistent with a lower risk of dementia after vaccination, but they do not establish that a specific herpesvirus initiates AD. Some participants were likely to have preclinical AD pathology at the time of vaccination, and a reduction in dementia diagnoses could reflect delayed progression, prevention of VZV-related vascular or inflammatory injury, or broader immunological effects.

Associations with non-herpesviruses have also been reported. A recent meta-analysis found higher odds of AD among individuals with hepatitis C virus (HCV) or severe acute respiratory syndrome coronavirus 2 (SARS-CoV-2) infection [36]. In a large electronic health-record cohort, COVID-19 was associated with an increased incidence of a new AD diagnosis within 360 days [41]. This interval is short relative to the preclinical phase of AD. A later UK Biobank study found no significant increase in AD risk after COVID-19. The excess dementia risk relative to uninfected controls was driven mainly by vascular dementia and was not greater than that observed after other respiratory illnesses [42]. The findings could therefore reflect accelerated clinical expression of pre-existing disease, vascular or inflammatory injury, increased healthcare contact, or diagnostic coding. Antiviral treatment for HCV has also been associated with a lower incidence of dementia [43]. However, the reduction was also observed among individuals who did not achieve sustained virological response, weakening the argument that the association was explained by viral clearance.

## 7. Viral Detection in PD

Viral infections have been linked to PD through epidemiological associations, experimental models, and reports of parkinsonism following viral encephalitis [3,44]. The long prodromal phase of PD complicates the temporal interpretation of infections recorded before diagnosis. Acute or postencephalitic parkinsonism demonstrates that viral infection can affect the nigrostriatal system but does not establish that the same viruses initiate idiopathic PD. No specific virus has been shown prospectively to precede the earliest detectable PD-related changes.

Chronic viral hepatitis has also been examined in relation to PD. Meta-analyses have found a modestly increased risk of PD in HCV-infected populations [36,45]. Results for HBV are inconsistent, with one recent meta-analysis reporting lower pooled odds in studies reporting odds ratios, but higher pooled hazards in studies reporting hazard ratios [45]. A register-based study also associated antiviral treatment for HCV with lower PD incidence [46]. However, the study lacked information on HCV severity, virological response, liver function, and lifestyle factors, and residual differences between treated and untreated patients cannot be excluded. For SARS-CoV-2, a large electronic health-record study found a transient increase in new PD diagnoses during the first year after infection, with no increase after 12 months [47]. This pattern may reflect unmasking of prodromal PD, post-infectious or vascular effects, or increased clinical surveillance.

Experimental studies show that viral infection can induce neuroinflammation, alter α-synuclein biology, and increase the vulnerability of dopaminergic neurons [3,48,49,50]. These effects provide possible mechanisms through which infection could contribute to PD initiation or progression. Most models involve acute infection or direct viral exposure, and their relevance to the slowly evolving human disease remains uncertain.

In a small exploratory postmortem study, human pegivirus (HPgV) RNA was detected in brain tissue from 5 of 10 individuals with PD and none of 14 controls. Detection by virome sequencing was confirmed by qPCR targeting two genomic regions, and HPgV nonstructural protein 5A immunoreactivity was demonstrated in oligodendrocytes in two HPgV-positive PD brains [8]. HPgV-positive individuals had more advanced tau neurofibrillary tangle pathology and higher complexin-2 levels, while Lewy body staging did not differ according to HPgV status. Exploratory transcriptomic analyses suggested differences in immune signaling, including suppression of interleukin-4 (IL-4)-related pathways, in HPgV-positive PD samples [8]. However, the brain transcriptomic analysis included only three HPgV-positive PD samples, and no individual genes remained significant after correction for multiple comparisons. The pathway findings should therefore be regarded as hypothesis-generating.

Longitudinal blood analyses provide information about peripheral HPgV infection. Persistence and clearance were observed in both PD and non-PD participants, with no clear disease-related pattern, although longitudinal data were available only for 11 HPgV-positive participants [8]. Associations between HPgV titer and immune signaling differed according to leucine-rich repeat kinase 2 (LRRK2) genotype, although these analyses included few individuals [8]. The study did not relate viral dynamics to subsequent clinical progression or PD-associated biomarkers.

The association with more advanced tau pathology does not show whether HPgV contributed to the pathology or became detectable in individuals with more extensive disease. Confirmation by virome sequencing, qPCR, and immunohistochemistry reduces the likelihood of a single assay artifact, but contamination or batch effects cannot be fully excluded in such a small tissue series. Active viral replication was not directly demonstrated, and the timing of CNS infection remains unknown [8]. The cerebral finding has not been independently replicated. HPgV is therefore best regarded as a candidate virus–disease association.

## 8. Viral Findings in ALS

Evidence for viral involvement in ALS is limited and inconsistent. A recent comprehensive review published in 2025 could not perform a meta-analysis of ALS because the available studies were too few. Among the exogenous viruses assessed, enterovirus was the only association reported in more than one study [36]. Enteroviral RNA has been detected in cerebrospinal fluid or CNS tissue in some studies, whereas others have not confirmed these findings [51]. Since the samples were obtained after symptom onset or at autopsy, detection does not show that infection preceded motor neuron degeneration. Human endogenous retrovirus K (HERV-K) expression has also been studied in ALS. An analysis of almost 2000 cortex and spinal cord RNA-sequencing samples found increased transcription of specific HERV-K loci in ALS spinal cord and identified a subgroup with increased expression in both regions [52]. These endogenous retroviral sequences are inherited genomic elements, however, and their expression is not evidence of an acquired viral infection. The available data cannot determine whether HERV-K expression contributes to motor neuron injury or results from altered transcriptional control in established disease. Table 1 summarizes the evidence and unresolved questions for the principal viral associations.

## 9. Interpreting Antiviral Immune Responses

Antiviral immune measurements primarily characterize the host response. Antibody concentrations are influenced by time since infection, cumulative antigen exposure, host genetics, age, immune function, and treatment. High concentrations may accompany recurrent antigen exposure or impaired viral control but cannot distinguish between these possibilities. The inferences supported by commonly used virological and antiviral immune measurements are summarized in Table 2.

Virus-specific T-cell responses depend on both antigen exposure and host immune function. An expanded response may reflect recent or recurrent antigenic stimulation or effective viral control. A weak response may reflect low antigen burden, immunosenescence, functional exhaustion, or treatment-related suppression [15]. Measurements of cytokine production, proliferation, or cytotoxicity provide information about T-cell function but do not determine whether an altered response preceded the neurological disease. Cross-sectional differences after diagnosis may have arisen from the disease, its treatment, or changes in viral activity. Interferon-related or inflammatory transcriptional profiles are not specific to antiviral immunity, since similar pathways may be activated by sterile tissue injury and protein aggregation [53,54].

The compartment in which the response is measured is also important. Virus-specific antibodies in cerebrospinal fluid may have entered from the circulation or been produced intrathecally. Paired serum and cerebrospinal fluid measurements are needed to distinguish intrathecal synthesis from passive transfer. Even when present, intrathecal production indicates a compartmentalized immune response and does not establish active viral replication. The polyspecific intrathecal antibody response to common viruses in MS illustrates that virus-specific antibodies can occur in the CNS without evidence that each recognized virus is involved in the disease [55]. Peripheral antiviral responses likewise provide limited information about viral activity within the brain.

A pathogenic role for an antiviral response requires evidence linking it to subsequent tissue injury. Cross-reactivity between viral and CNS antigens, virus-specific cytotoxicity, or cytokine-mediated injury can establish a plausible mechanism [20,56]. Their relevance to human disease depends on whether the implicated immune response is present before or during the corresponding pathological change.

## 10. Studies Needed to Distinguish Viral Roles

Prospective cohorts with archived serial samples can establish whether primary infection or changes in viral activity precede disease-associated biomarkers and clinical onset. For EBV and MS, this may include initially seronegative individuals in whom seroconversion can be identified [6]. For herpesviruses usually acquired early in life, studies of AD will generally need to focus on reactivation or changes in viral control during the preclinical and clinical phases [12]. In PD, prospective studies can examine whether persistent or newly detected viral activity precedes biomarker changes or clinical progression [13].

Within longitudinal cohorts, repeated blood or saliva measurements can be related to subsequent imaging findings, disease biomarkers, and clinical progression. Sampling before and after treatment initiation can identify treatment-related changes in viral control. Paired blood and cerebrospinal fluid samples may be feasible in smaller nested studies. Comparative meta-analyses that evaluate multiple viruses and neurodegenerative diseases can show how pooled estimates vary across the associations examined [36]. Their ability to address specificity is limited, however, by differences in study design, exposure assessment, outcome definition, and confounder adjustment. Within individual cohorts, comparison viruses and neurological control groups remain important for determining whether changes are virus-specific or accompany neurological disease more generally.

Brain tissue studies provide complementary evidence. Viral findings should be replicated in independent collections and confirmed using more than one detection method. Cellular localization, markers of viral replication, and the spatial relationship between viral signals and disease pathology can help distinguish active infection from incidental viral material. Longitudinal brain donation cohorts offer an opportunity to relate postmortem findings to antemortem clinical and biomarker data, although such samples will remain limited [57].

Ultimately, intervention studies are most informative when they document the intended effect on the proposed viral process and assess a subsequent disease-related outcome. A reduction in viral activity alone does not show that the intervention has altered the neurological disease. Trials of EBV vaccination can initially assess prevention of infection or infectious mononucleosis, with longer-term follow-up for MS [58]. Studies of zoster vaccination can incorporate measures of VZV reactivation and biomarker substudies addressing amyloid, tau, and neurodegeneration [7,39,40]. For HPgV, cerebral detection should first be replicated and shown to represent local viral activity. Large pooled longitudinal PD and prodromal cohorts with archived samples would be needed to identify sufficient numbers of HPgV-positive participants before peripheral viral activity could be related meaningfully to α-synuclein biomarkers or clinical progression [8,59].

## 11. Conclusions

Viral associations in neurodegenerative diseases should be interpreted according to when infection or viral activity occurs in relation to the disease process. Considering disease initiation, modification of established disease, impaired viral control secondary to disease or treatment, and incidental detection as separate interpretations identifies the temporal evidence and measurements needed to evaluate each one. Evidence strongly supports an upstream role for EBV in MS development. Findings in AD, PD, and ALS remain compatible with several interpretations and generally lack comparable prospective temporal evidence.

Priorities are longitudinal studies that link repeated virological and immune measurements to subsequent disease biomarkers and clinical outcomes, independent confirmation of brain-tissue findings, and intervention studies that test whether preventing or suppressing the implicated viral process changes disease risk or progression.

## Figures and Tables

**Table 1:** Interpretation of the principal viral associations.

Association	Main Evidence	Temporal Information	Supported Conclusion	Main Unresolved Question
EBV and MS	Prospective seroconversion, serum NfL, serology and mechanistic studies	EBV infection precedes the first observed NfL increase and clinical onset	Evidence strongly supports an upstream role for EBV in MS development	Whether ongoing EBV activity modifies established MS
HSV-1, HHV-6/7 and AD	Serology, postmortem detection and experimental models	Viral activity has not been prospectively placed before AD biomarkers	Experimental studies show that herpesviruses can interact with AD-associated proteins and pathways	Whether infection initiates, accelerates or follows AD pathology
VZV vaccination and dementia	Natural experiments and observational studies	Vaccination generally occurs when preclinical pathology may already be present	Zoster vaccination reduces the occurrence of dementia diagnoses, but the mediating pathway remains unresolved	Whether protection results from reduced VZV reactivation, broader immune effects or both
CMV, HCV and SARS-CoV-2 with AD or dementia	Serology, health-record cohorts, meta-analyses and antiviral-treatment cohorts	Exposure generally precedes diagnosis but has not been placed before the earliest AD biomarkers	Modest and heterogeneous associations have been reported, without establishing temporal direction or virus-specific effects	Virus-specific effects versus systemic or vascular illness, comorbidity, pre-existing disease and ascertainment
HBV, HCV and SARS-CoV-2 with PD	Epidemiological cohorts and meta-analyses; antiviral-treatment studies for HCV	Infection or diagnosis precedes PD diagnosis, but not the earliest PD-related changes; associations following SARS-CoV-2 appear transient	HCV has been associated with a modest increase in PD risk. Findings for HBV are inconsistent. Increased PD diagnoses following SARS-CoV-2 have been confined mainly to the first year	Whether the associations reflect disease initiation, systemic effects, unmasking of prodromal PD, or increased clinical ascertainment
HPgV and PD	Viral RNA and protein in a small postmortem series and longitudinal blood measurements	Cerebral detection occurred in established disease	HPgV is a candidate virus-disease association	Reproducibility, local replication and temporal direction
Enteroviruses and HERV-K in ALS	Case–control viral detection studies and postmortem RNA sequencing	Measurements obtained after symptom onset or at autopsy	Enterovirus findings are inconsistent. Locus-specific HERV-K dysregulation occurs in a subset of ALS tissue	Whether either finding precedes or contributes to motor neuron degeneration

**Table 2:** Interpretation of virological and antiviral immune measurements.

Measurement	What It Can Establish	What It Cannot Establish
Seroconversion	Approximate timing of primary infection	That infection initiated the disease
Seropositivity	Previous infection	Timing, reactivation or current viral burden
Antibody concentration	Magnitude of the host antibody response	Viral load or the reason for an altered response
Viral load or shedding	Quantity or release of viral material in the sampled compartment	Replication, CNS involvement, or direction of association, depending on the virus and assay
Viral nucleic acid or protein in brain tissue	Presence of viral material	Replication, infected cell type or disease relevance
Replication markers with cellular localization	Local viral activity and involved cell type	Whether infection preceded or contributed to pathology
Virus-specific T-cell responses	Frequency or function of responding T cells	Whether the response is protective, pathogenic or secondary
Intrathecal virus-specific antibodies	A compartmentalized response when intrathecal synthesis is demonstrated	Active CNS infection or pathogenic involvement
Interferon or inflammatory transcriptional profiles	Activation of immune pathways	Virus-specific immune activation
Targeted intervention	Whether the intervention changes the proposed viral process and a subsequent disease-related outcome	That any clinical effect is mediated by the virus when the intervention has broader biological effects

## Data Availability

No new data were created or analyzed in this study. Data sharing is not applicable to this article.
